# Expression of IL-33 in ocular surface epithelium induces atopic keratoconjunctivitis with activation of group 2 innate lymphoid cells in mice

**DOI:** 10.1038/s41598-017-10227-y

**Published:** 2017-08-30

**Authors:** Yasutomo Imai, Yuka Hosotani, Hiroto Ishikawa, Koubun Yasuda, Makoto Nagai, Orie Jitsukawa, Fumi Gomi, Kenji Nakanishi, Tomohiro Yoshimoto, Takahiro Nakamura, Kiyofumi Yamanishi

**Affiliations:** 1 Department of Dermatology, Hyogo College of Medicine, Nishinomiya, 663-8501 Hyogo Japan; 2 Department of Ophthalmology, Hyogo College of Medicine, Nishinomiya, 663-8501 Hyogo Japan; 3 Department of Immunology, Hyogo College of Medicine, Nishinomiya, 663-8501 Hyogo Japan; 4 Department of Frontier Medical Science and Technology for Ophthalmology, Kyoto Prefectural University of Medicine, Kamigyo-ku, 602-0841 Kyoto, Japan

## Abstract

In a transgenic mouse line hK14mIL33tg, with the expression of interleukin-33 (IL-33) driven by a keratin 14 promoter, keratoconjunctivitis developed spontaneously between 18 and 22 weeks of age under specific-pathogen-free conditions. These mice showed blepharitis and corneal impairments, and the histology revealed epithelial thickening in the conjunctiva and the cornea with infiltration of eosinophils, mast cells and basophils. IL-5, IL-13 and CCL11 were abundant in lacrimal fluid in the mice, and the gene expressions of IL-4, IL-5, IL-13, IL-33, Prg2 and Mmcp8 were significantly increased in the cornea. Furthermore, group 2 innate lymphoid cells (ILC2) producing IL-5 and IL-13 were markedly increased in the cornea. These phenotypes closely resemble human atopic keratoconjunctivitis (AKC). The characteristic ocular phenotype in these mice strongly suggests that IL-33 is crucial for the development of AKC. The mouse line may be useful as a novel model for research and development of therapeutic strategies for AKC.

## Introduction

Interleukin-33 (IL-33), a pro-inflammatory cytokine of the IL-1 family^1^, is localized and stored in nuclei of epithelial cells in steady state^2^. However, in response to various stimuli or conditions, IL-33 is released as an alarmin^2, 3^ and, via binding to its receptor ST2 (IL-1RL1), activates innate immune cells, such as group 2 innate lymphoid cells (ILC2) (previously termed natural helper cells)^4^, basophils and mast cells^5^, with release of Th2-type cytokines IL-4, IL-5 and/or IL-13^4, 5^. In accordance with these immunological characteristics, the involvement of IL-33 has been suggested in the pathophysiology of allergic disorders, asthma^5^, allergic rhinitis^6^, atopic dermatitis (AD)^7, 8^ and urticaria^9^. Because IL-33 is constitutively expressed in the nuclei of conjunctival epithelial cells, and because exogenous IL-33 augments ragweed-induced allergic conjunctivitis in mice^10^, IL-33 may also mediate in allergic conjunctivitis. However, in contrast with this acquired immunity model, eyedrop administration of exogenous IL-33 alone failed to induce conjunctivitis in unimmunized animals^10^.

Atopic keratoconjunctivitis (AKC) is a chronic inflammatory disorder of the ocular surface that occurs mainly in AD patients with facial involvement. Conjunctival inflammation with eosinophil infiltrates in AKC, sometimes extending to the cornea and leading to corneal ulcer, scar formation and, ultimately, to vision loss^11, 12^. IL-33 is indeed up-regulated in the conjunctival tissues of AKC^13^, but precise mechanisms of how IL-33 contributes to those inflammatory processes and the role of ILC2 in AKC are still unknown.

Recently, we established a transgenic mouse line expressing a mouse IL-33 gene driven by a keratin 14 promoter. In these mice, AD-like itchy dermatitis developed spontaneously with induction of ILC2 in lesional skins^7^. In further observing mouse phenotypes, we noticed that severe keratoconjunctivitis occurred with age in these mice. Interestingly, the eye lesions accompanying the activation of ILC2 and the phenotype strikingly resembled human AKC. In the present study, based on these observations, we demonstrate that the up-regulation of IL-33 in the ocular surface epithelium causes AKC *in vivo*.

## Results

### Spontaneous development of keratoconjunctivitis in hK14mIL33tg (IL33tg), transgenic mice with keratin 14-driven expression of IL-33

**Figure 1:**
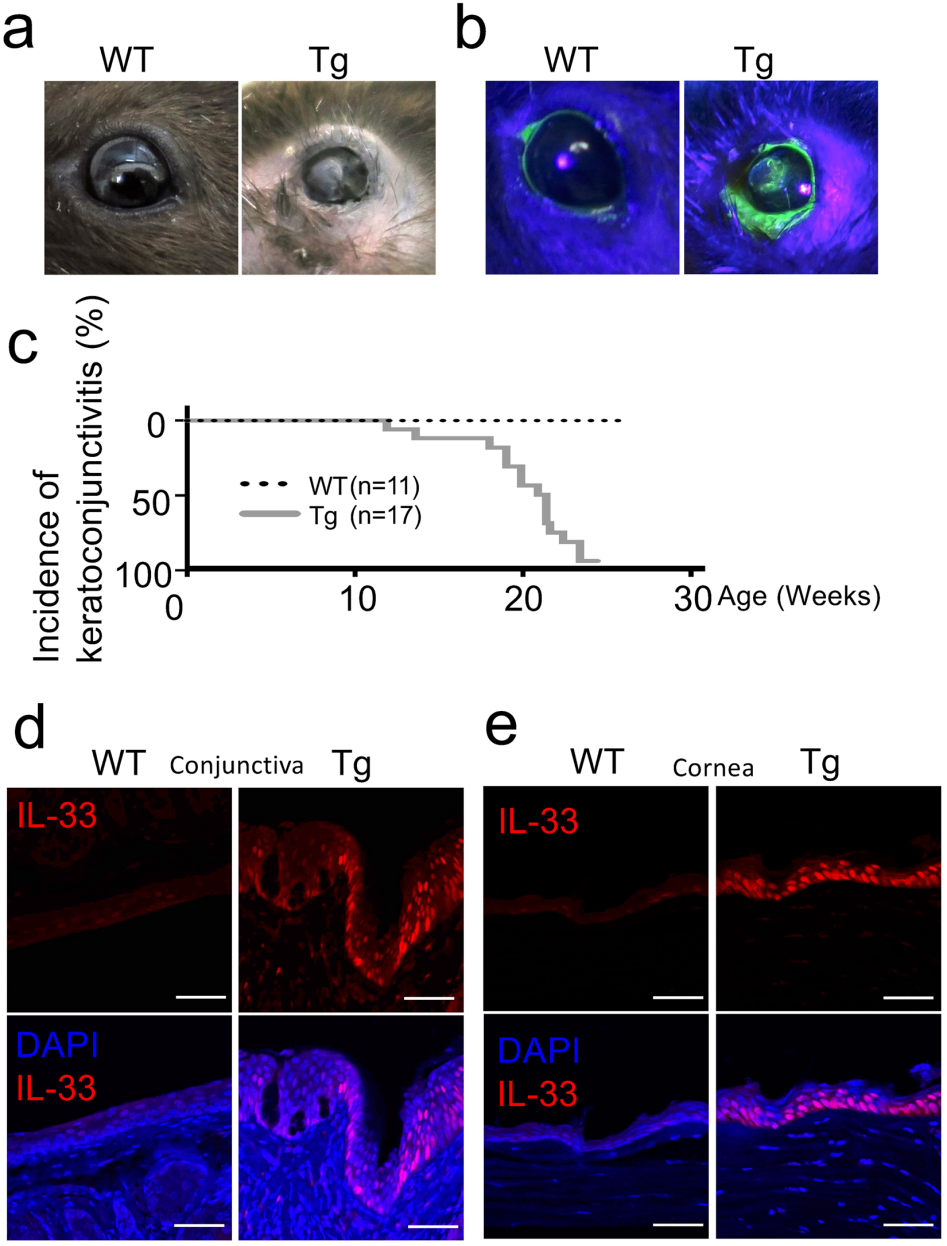
Spontaneous development of atopic keratoconjunctivitis (AKC)-like lesions in hK14mIL33tg (IL33tg) mice. (**a**,**b**) Keratoconjunctival manifestations (**a**) and fluorescein staining of cornea (**b**) of wild-type (WT) and hK14mIL33tg (IL33tg) mice (Tg). Representative photographs taken from at least six 24- to 30-week-old mice are shown. Similar keratoconjunctival lesions were observed in three independent experiments. (**c**) Incidence of spontaneous keratoconjunctivitis that develops in IL33tg mice. Keratoconjunctivitis developed spontaneously between 18 and 22 weeks of age and remained thereafter in all IL33tg mice. WT mice (n = 11), IL33tg mice (Tg) (n = 17). P < 0.0001 by the two-tailed log-rank test. (**d**,**e**) Immunofluorescence of IL-33 in the ocular surface epithelium of WT and IL33tg mice (Tg). Intense staining of IL-33 was evident in nuclei of the conjunctival epithelium (**d**) and corneal epithelial cells (**e**) in 20- to 30-week-old IL33tg mice. Bars, 50 µm. Data are representative of three independent experiments.

In a transgenic mouse line hK14mIL33tg (IL33tg), the expression of the mouse IL-33 gene (*Il33*) is driven by a human keratin 14 (K14) promoter^7^. Almost all IL33tg mice spontaneously developed eye lesions under specific-pathogen-free (SPF) conditions. IL33tg mice showed blepharitis (Fig. 1a), shield ulcer-like corneal ulcer, corneal opacity, corneal epithelial defect and corneal plaque formation (Fig. 1a,b). Fluorescein staining, which was used to indicate the area of epithelial damage, was clearly positive in the center of the cornea in IL33tg mice (Fig. 1b). The eye lesions occurred between 18 and 22 weeks of age, later than AD-like dermatitis that became grossly visible between 6 and 8 weeks of age (Fig. 1c).

Since K14 is expressed in human or mouse conjunctival, corneal and limbal epithelia^14^, the expression of IL-33 in those epithelia of IL33tg mice was examined by immunohistochemistry. As shown in Fig. 1 d and e, IL-33 was strongly positive in the nuclei of conjunctival (Fig. 1d) and corneal (Fig. 1e) epithelia of IL33tg mice, compared with wild-type (WT) mice. A low magnification overview of the eyelid is shown in Supplementary Fig. 1.

### Infiltration of eosinophils and mast cells into conjunctiva and cornea of 20- to 30-week-old IL33tg mice

**Figure 2:**
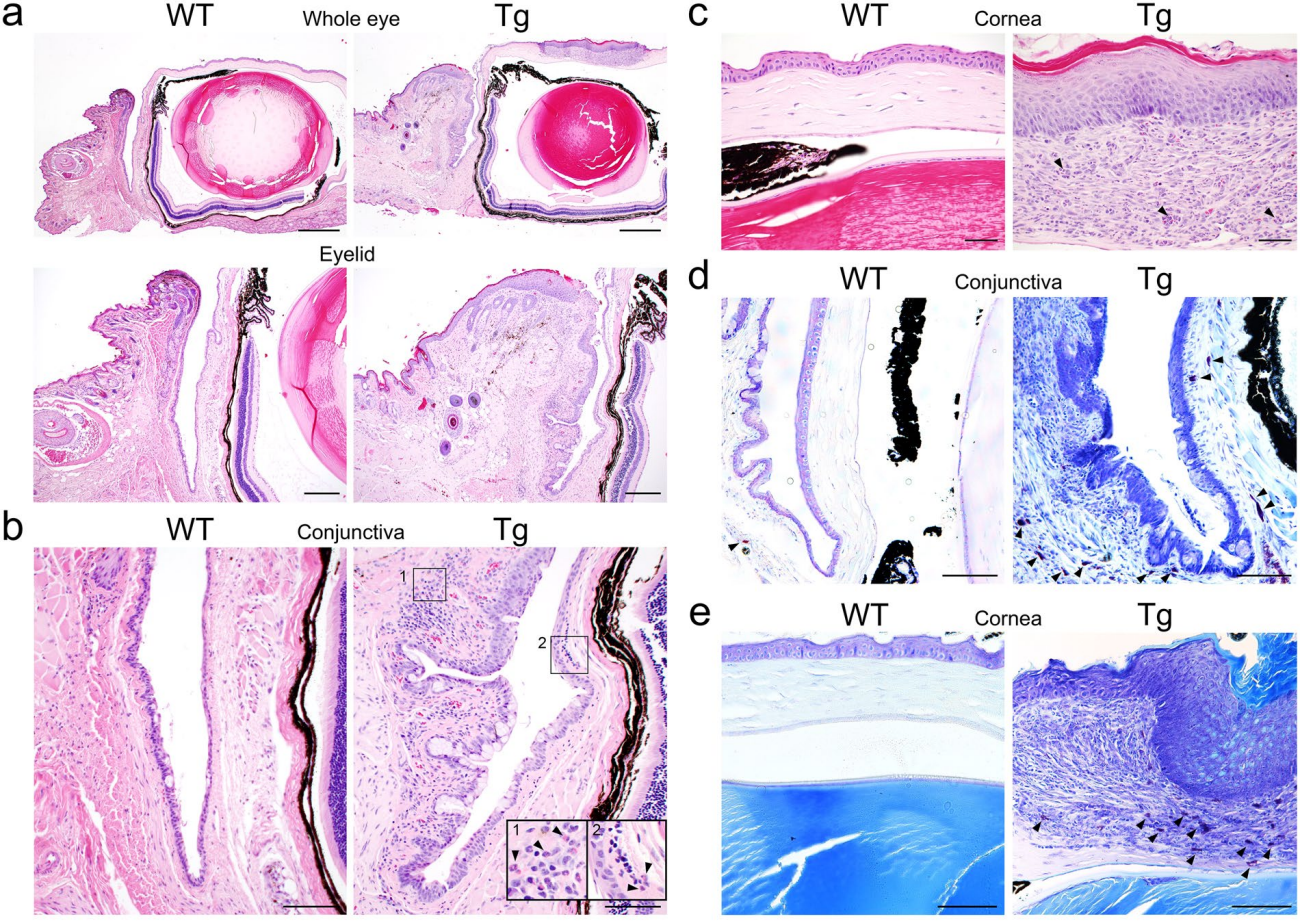
Keratoconjunctivitis with eosinophil infiltrates in 20- to 30-week-old IL33tg mice. (**a**–**c**) H&E staining of WT and IL33tg mouse (Tg) conjunctiva and cornea. Cell infiltrations including eosinophils (arrowheads) were evident in the conjunctiva (**b**) and the corneal stroma (**c**) of IL33tg mice. Panels are representative of six mice and of three independent experiments. (**a**) The whole eye (upper panels). Bars, 500 µm; the eyelid (lower panels). Bars, 200 µm. (**b**) The conjunctiva. Bars, 100 µm. (**c**) The cornea. Bars, 50 µm. (**d**,**e**) Toluidine blue staining of WT and IL33tg mouse (Tg) conjunctiva and cornea. Toluidine blue-positive mast cells (arrowheads) were abundant in the conjunctiva and the corneal stroma of IL33tg mice. (**d**) The conjunctiva. Bars, 100 µm. (**e**) The cornea. Bars, 100 µm. Panels are representative of at least three mice and two independent experiments.

In hematoxylin and eosin (H&E) staining (Fig. 2a–c), eosinophil infiltration (Fig. 2b, arrowheads) with hyperplasia of goblet cells was evident in the conjunctiva (Fig. 2b). The corneal epithelium thickened, and inflammatory cells infiltrated the lesions of IL33tg mice (Fig. 2a,c), compared with an intact epithelium in WT mice. Abundant toluidine blue-positive mast cells, possibly connective tissue mast cells^15, 16^, were seen in the conjunctival lesions of IL33tg mice (Fig. 2d, arrowheads) with some degranulating mast cells. The infiltration of mast cells was also evident in the corneal stroma of IL33tg mice (Fig. 2e, arrowheads). These histological features are comparable with AKC regarding the infiltration of eosinophils and mast cells.

### Increased Th2 cytokines and chemokines in the lacrimal fluid of 20- to 30-week-old IL33tg mice

**Figure 3:**
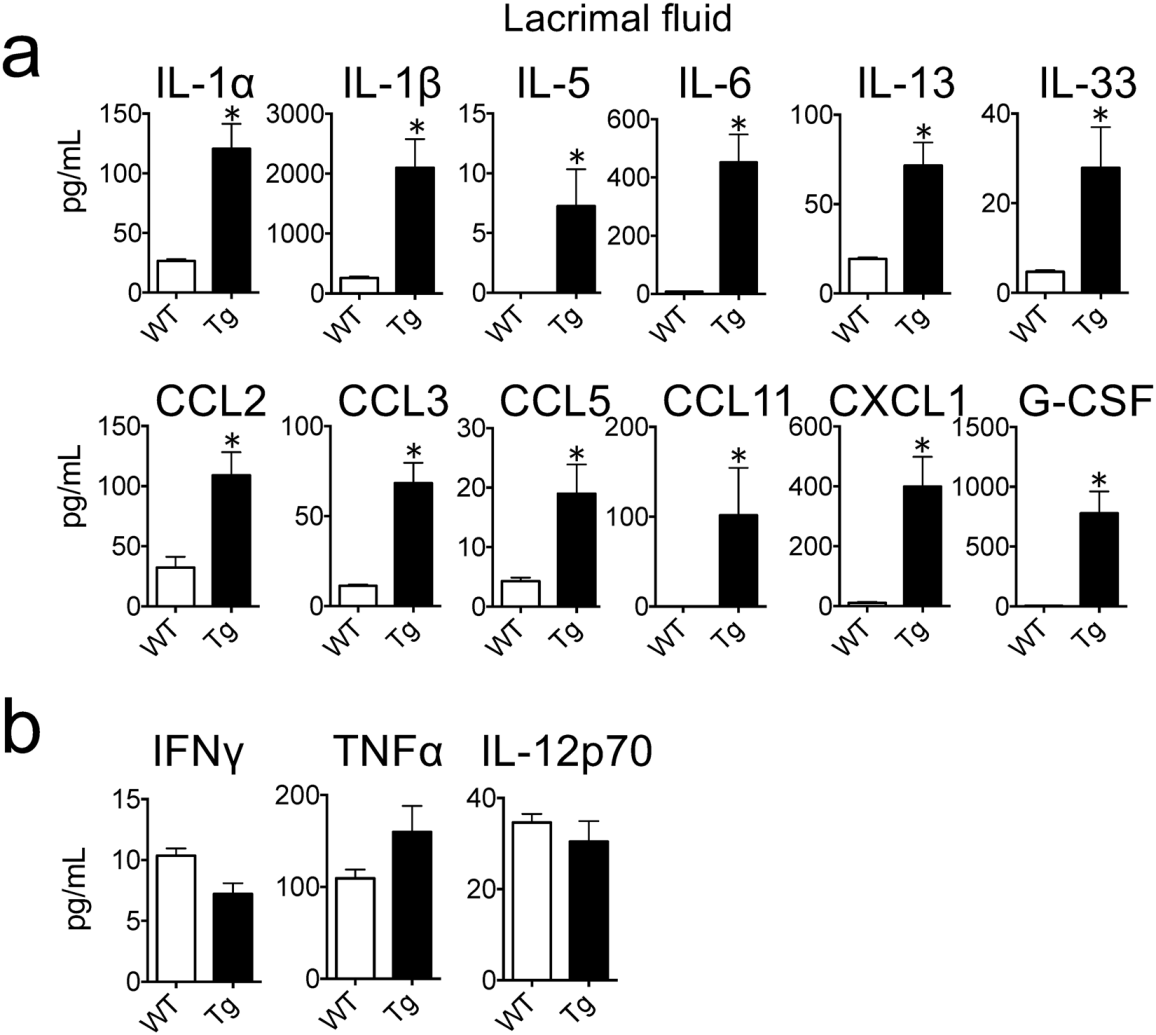
Cytokine and chemokine profiles of lacrimal fluid in 20- to 30-week-old IL33tg mice. The concentrations of cytokines and chemokines in lacrimal fluid of 20- to 30-week-old WT and IL33tg mice (Tg) were measured as described in Materials and Methods. IL-5, IL-13, CCL5 (RANTES) and CCL11 (Eotaxin-1) were increased (**a**), whereas IFN-γ, TNF-α and IL-12p70 were unaltered (**b**) in the lacrimal fluid of IL33tg mice. Data are expressed as means ± SEM (n = 14), *P < 0.05 (Mann-Whitney test).

Cytokines and chemokines in the lacrimal fluid of IL33tg mice were examined using a protein array (Fig. 3). The concentration of IL-33 in the lacrimal fluid was much higher in IL33tg mice than in WT mice. IL-1α, IL-1β, IL-5, IL-6, IL-13, CCL2, CCL3, CCL5, CCL11, CXCL1 and G-CSF were also significantly higher in the lacrimal fluid of IL33tg mice (Fig. 3a). On the other hand, Th1 cytokines such as IFN-γ, TNF-α and IL-12p70 were unaltered (Fig. 3b). The lacrimal fluid cytokine and chemokine profile with the infiltration of eosinophils in IL33tg mice coincides with that of patients with AKC^17–19^. The infiltration of eosinophils in the lesions may be associated with Th2 cytokines and chemokines such as IL-5, IL-13, CCL5 and CCL11.

### Gene expression of Th2 cytokines and markers for eosinophils and basophils in 20- to 30-week-old IL33tg mouse conjunctiva and cornea

**Figure 4:**
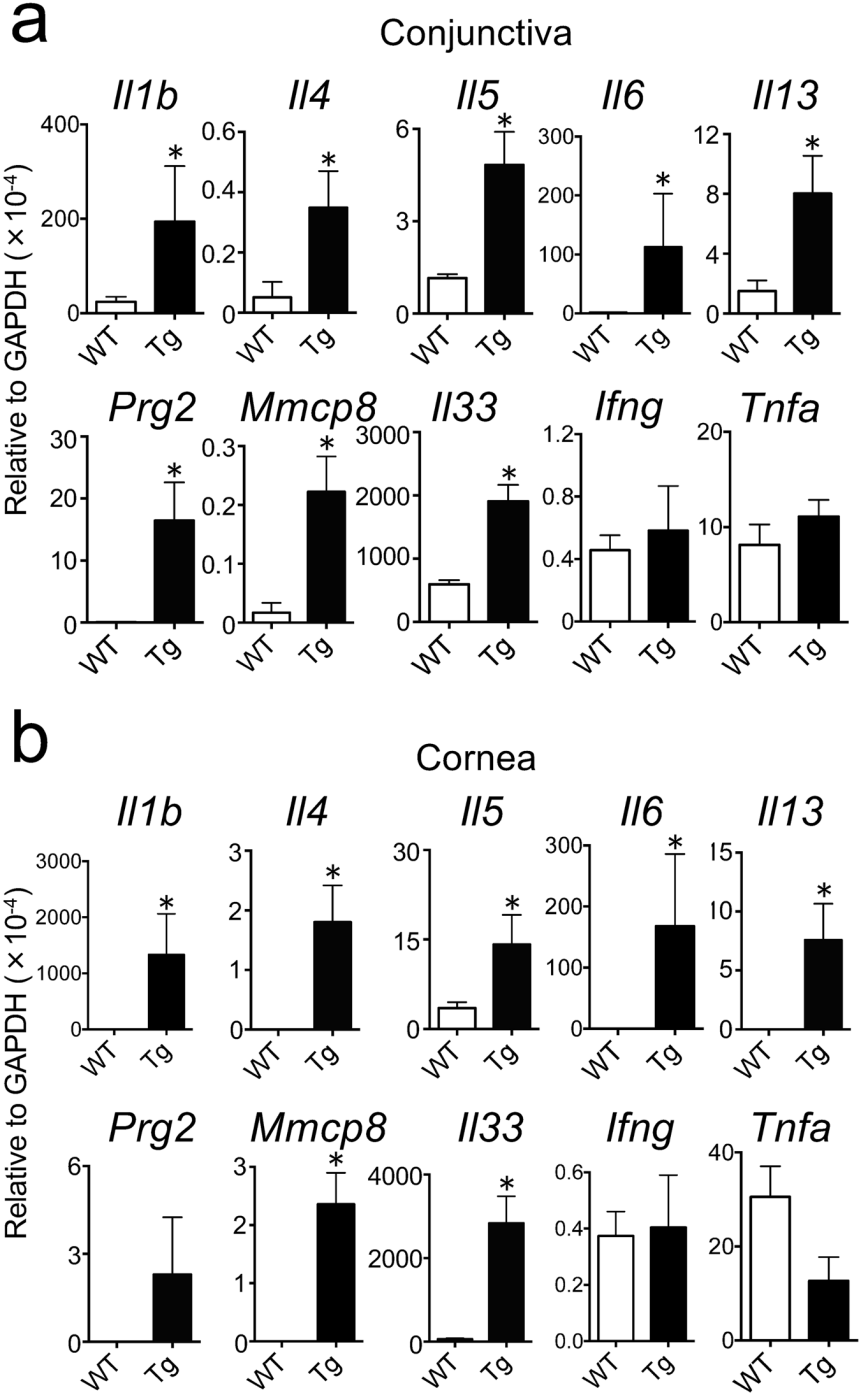
The gene expressions of Th2 cytokines, eosinophil granule major basic protein (Prg2) and mouse mast cell protease 8 (Mmcp8) in the conjunctiva and cornea of 20- to 30-week-old IL33tg mice. Note that *Il4*, *Il5*, *Il13*, *Prg2*, *Mmcp8* and *Il33* were significantly increased in the conjunctiva (**a**) and cornea (**b**) of IL33tg mice (Tg). Data are expressed as means ± SEM (n = 7 (**a**) or n = 8 (**b**)), *P < 0.05 (Mann-Whitney test).

The gene expression of IL-33 and Th2 cytokines in mouse conjunctiva and cornea were examined using quantitative real-time polymerase chain reaction (qPCR). As shown in Fig. 4a, the expression of *Il33* in the conjunctiva was significantly higher in IL33tg mice than in WT mice, and *Il4*, *Il5*, and *Il13* were also significantly increased in the tissue of IL33tg mice versus that of WT mice. Eosinophil granule major basic protein (Prg2) is a marker for eosinophils; basophil-specific granzyme B-like protease (Mmcp8) is the first lineage‐specific differentiation marker for mouse basophils. The gene expressions of *Prg2* and *Mmcp8* in the conjunctiva were also significantly higher in IL33tg mice than in WT mice.

Similarly, the expressions of *Il33*, *Il4*, *Il5*, *Il13, Prg2* and *Mmcp8* were markedly increased in the corneas of IL33tg mice (Fig. 4b). These results may reflect the infiltration of eosinophils and basophils in the ocular tissues of IL33tg mice.

### Induction of ILC2 in 20- to 30-week-old IL33tg mouse cornea

**Figure 5:**
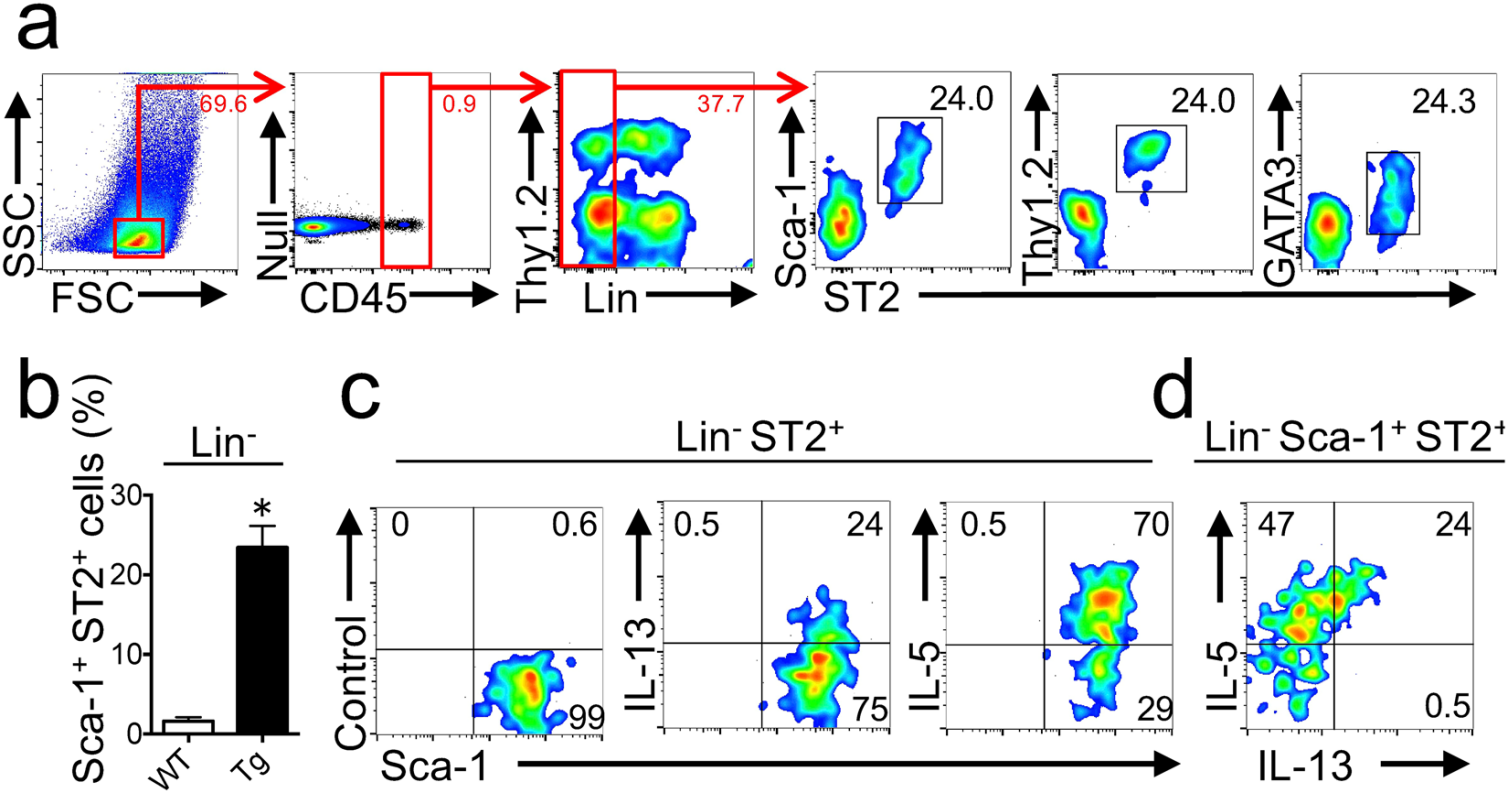
Induction of ILC2 in IL33tg mouse cornea. (**a**) Gating strategy for analysis of FSC^low^SSC^low^CD45^+^Lineage(Lin)^−^Sca-1^+^ST2^+^ ILC2 cells. Note that ILC2 cells are Thy1.2^+^ and GATA3^+^. The numbers indicate the percentage of cells in each gate. (**b**) The proportion of Sca-1^+^ ST2^+^ ILC2 from the corneas of 20- to 30-week-old WT and IL33tg mice (Tg) determined by flow cytometry. Values indicate the percentage of ILC2 of Lin^−^ lymphocytes. Data are expressed as means ± SEM (n = 3) *P < 0.0001 (two-tailed *t*-test). (**c**,**d**) The intracellular flow cytometry for IL-5 and IL-13. The cells were gated into CD45^+^Lin^−^ fraction (**c**) and CD45^+^Lin^−^Sca-1^+^ST2^+^ fraction (d). The numbers indicate the percentage of cells in each quadrant. Similar results were obtained in two (**a**,**b**) or three (**c**,**d**) independent experiments.

Concentrations of IL-5 and IL-13 were abundant in the lacrimal fluid, and the gene expression of these cytokines in the cornea was strongly induced in IL33tg mice. This suggests that ILC2 increase ocular surface lesions, as these Th2 cytokines are massively produced by ILC2^4^. We next examined lineage-markers (Lin)^−^ST2^+^Sca-1^+^ ILC2 in the cornea using flow cytometry (Fig. 5a). These corneal ILC2 cells were surface Thy1.2^+^ and intracellular GATA3^+^. While ILC2 resided in the corneas of WT mice, these cells were more abundant in the corneas of IL33tg mice (Fig. 5b). The proportion of ILC2 in the corneal cells was about 20 times greater in IL33tg mice than in WT mice. To determine the cells producing Th2 cytokines, corneal cell suspensions from IL33tg mice were subjected to intracellular staining for IL-5 and IL-13 (Fig. 5c). Lin^−^Sca-1^+^ST2^+^ ILC2 from IL33tg mouse cornea expressed high levels of IL-5 and IL-13. Interestingly, IL-13-producing ILC2 cells also produced IL-5 (Fig. 5d). In addition, almost all cells expressing IL-5 or IL-13 were Lin^−^ST2^+^Sca-1^+^ ILC2 (Supplementary Fig. 2a,b), and Lin^+^ cells did not produce Th2 cytokines (Supplementary Fig. 2a). These results suggest that ILC2 are a major source for IL-5 and IL-13 in the lesional corneas of IL33tg mice.

### Induction of basophils in 20- to 30-week-old IL33tg mouse cornea

**Figure 6:**
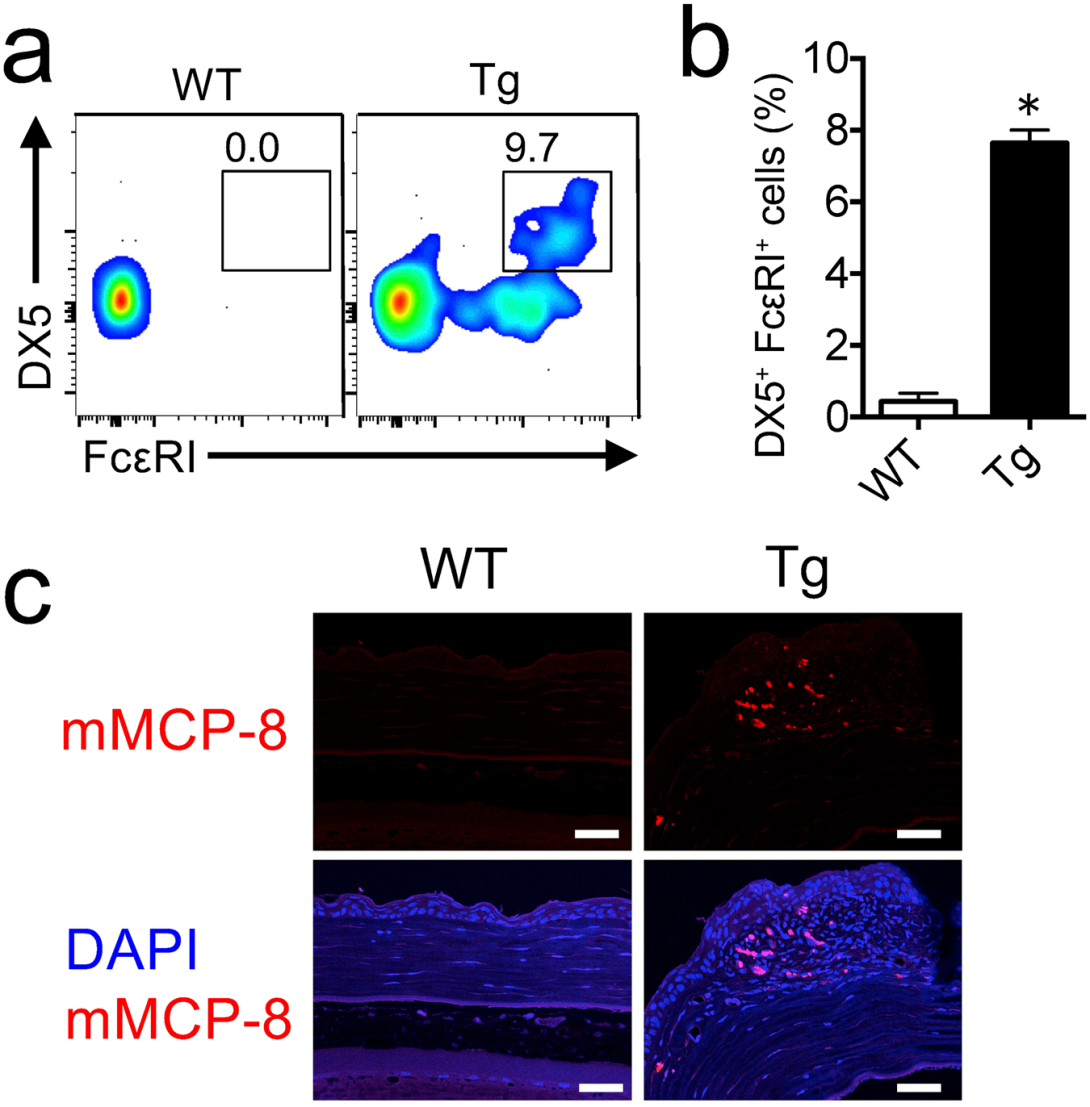
Induction of basophils in IL33tg mouse cornea. (**a**) Flow cytometry of cells from the corneas of 20- to 30-week-old WT and IL33tg mice (Tg). Cells were gated on the B220^−^CD3^−^CD4^−^CD8^−^Gr-1^−^NK1.1^−^Siglec-F^−^CD45^+^ fraction. The numbers indicate the proportion of DX5^+^FcεR1^+^ cells. (**b**) The proportion of DX5^+^FcεR1^+^ determined by flow cytometry. Data are expressed as means ± SEM (n = 3) *P < 0.01 (two-tailed *t*-test). (**c**) Immunofluorescence of mMCP-8 in the corneal stroma of WT and IL33tg mice (Tg). The infiltration of mMCP-8^+^ basophils were evident in the corneal stroma of IL33tg mice. Bars, 50 µm. Data are representative of at least six mice. Similar results were obtained in two independent experiments.

The increase of *Mmcp8*, a marker for mouse basophils, in IL33tg mouse corneas prompted us to examine basophil infiltration in the cornea. Flow cytometry showed that DX5^+^FcεRI^+^ basophils were significantly increased in the corneal cells of IL33tg mice, compared with WT mice (Fig. 6a,b). Immunohistochemistry revealed that mMCP-8^+^ basophils were abundant in the corneal stroma of IL33tg mice (Fig. 6c).

### Conjunctivitis precedes keratitis in IL33tg mice

Compared with WT mice, IL-33 was strongly positive in the nuclei of conjunctival (Supplementary Fig. 3a) and corneal (Supplementary Fig. 3b) epithelia in 8- to 10-week-old IL33tg mice. In these IL33tg mice, conjunctivitis with eosinophilic infiltration (Supplementary Fig. 3c,e) was often evident, but keratitis was not observed (Supplementary Fig. 3d,f). The expressions of *Il4*, *Il5*, *Il13, Il33, Prg2* and *Mmcp8* were significantly increased in the conjunctiva (Supplementary Fig. 4a) in 10-week-old IL33tg mice versus WT mice, but not in the corneas (Supplementary Fig. 4b). These results suggest that conjunctivitis precedes keratitis in IL33tg mice.

The gene expressions of *Il1b* and *Il6* were not increased in the conjunctiva and cornea of 10-week-old IL33tg (Supplementary Fig. 4a), in contrast with a significant increase in 20- to 30-week-old IL33tg mice versus WT mice (Fig. 4a). Although the reason for the induction of these genes is unknown, it may reflect epithelial thickening associated with inflammation.

## Discussion

The K14 promoter is used for constitutive activation of genes in mainly stratified squamous epithelia, such as skin, parts of the respiratory, digestive and genital tracts, and ocular surface epithelium. We generated a transgenic mouse line, in which mouse IL-33 is overexpressed under the control of a K14 promoter, and characterized the skin phenotypes resembling AD^7^. We next focused on the ocular lesions spontaneously developed in the mice and elucidated the pathological effect of lifelong overexpression of IL-33 on conjunctiva and cornea.

In this study, we clearly demonstrate that the overexpression of IL-33 in conjunctival and corneal epithelia induces AKC-like inflammation *in vivo*. IL-33 is increased in the conjunctiva of AKC patients^13^, but the role of IL-33 in the pathogenesis of AKC had not been fully understood. Our present findings strongly suggest that IL-33 mediates the development of AKC.

Several mouse models for allergic conjunctivitis have been reported^10, 20, 21^, but an AKC model in which conjunctival inflammation extends to the cornea and keratitis develops had not been documented. In IL33tg mice, the infiltration of eosinophils, mast cells and basophils are evident in the conjunctival and corneal epithelia. This is comparable to the pathological findings in human AKC in which eosinophils^22, 23^, mast cells and basophils^21^ infiltrate the conjunctiva and the corneal stroma.

We have shown that IL-33 activates mast cells *in vitro* ^6^, mast cells accumulate in AD-like eruptions and serum IgE levels are high in IL33tg mice^7^. The phenotype of AKC-like lesions in IL33tg mice suggests that mast cells in keratoconjunctivitis are activated in response to IL-33, which is overexpressed in the ocular surface epithelium *in vivo*. The AKC lesions that develop spontaneously in IL33tg mice may be useful for research on the corneal inflammation associated with AD, since biopsy of the cornea is clinically difficult^23^ and since the pathophysiology of corneal involvement remains to be addressed in human AKC tissues.

In AKC patients, cytotoxic proteins from eosinophils and proteases from mast cells are postulated to damage the corneal epithelium^23, 24^. Subsequently, IL-4 and IL-13 penetrate the damaged sites of the epithelium and activate keratocytes to produce cytokines and/or chemokines such as CCL11^25^. Possibly, those molecules further recruit eosinophils and mast cells in the cornea. Th2 cytokines are dominant in the lacrimal fluids of IL33tg mice, in contrast to Th1 cytokines (Fig. 3). Previous reports showed increases in Th2 cytokines IL-5^18^ and IL-13^19^. CCL11, a chemokine, is also increased in the lacrimal fluids of AKC patients, and the concentration is especially high in AKC with corneal erosions^17^. Thus, the cytokine and chemokine profile in lacrimal fluids of IL33tg mice is largely comparable with that reported in human AKC, although it is still controversial whether IFN-γ, a Th1 cytokine, is increased in lacrimal fluids of AKC patients^11, 18, 26^, and the cytokine was not increased in lacrimal fluids of IL33tg mice.

The number of patients with allergic conjunctival diseases peaks in the early twenties, and the incidence decreases with age by spontaneous regression^12^. However, Ono *et al*.^27^ reported that AKC occurs more frequently between the ages of 30 and 50. The ocular surface inflammation in IL33tg mice does not subside with age, possibly because of sustained IL-33 expression in the epithelium, and therefore the keratoconjunctivitis occurred in IL33tg might be a chronic disease model for AKC.

In the acquired immunity model of allergic conjunctivitis^10^, we showed that exogenous IL-33 activates ragweed pollen-specific Th2 cells. AKC is suggested to be a T cell-mediated disease^11, 12^. However, ILC2, like T cells (Th2 cells), may play a role in keratoconjunctivitis in IL33tg mice, as ILC2 can produce massive IL-5 and IL-13 in response to the stimulation of IL-33^4^, and those Th2 cytokines are produced by ILC2 in the corneas of IL33tg mice (Supplementary Fig. 2). Interestingly, we found the presence of ILC2 in the intact corneas of WT mice (cornea-resident ILC2), but ILC2 were greater in the corneas of IL33tg mice (Fig. 5a,b). Furthermore, IL-5 and IL-13 in the cornea are produced only by CD45^+^Lin^−^Sca-1^+^ST2^+^ ILC2 (Supplementary Fig. 2). ILC2 residing naturally in peripheral tissues are now classified as “natural helper cells” or “natural ILC2 (nILC2)”^28^. They also have been identified in the lung respiratory epithelium^29^ and in the skin^7^.

The increased expression of basophil-specific *Mmcp8* and the infiltration of basophils in the cornea were demonstrated in IL33tg mice. We have reported that excess IL-33 causes migration of basophils via induction of CCL2, CCL3 and CCL5^6^. We therefore speculate that these chemokines, which were significantly increased in lacrimal fluids, might be involved in the recruitment of basophils into the corneas of IL33tg mice.

Inflammatory events elicited by IL-33 in the conjunctiva and cornea in IL33tg mice may be a complex process involving basophils and ILC2 in concert with activation of Th2 cytokines/chemokines. Basophil-produced IL-4 influences not only the expression of Th2 cytokines and chemokines in ILC2, but the cellular expansion of ILC2^30^; IL-33-stimulated ILC2 produce CCL5 and CCL11, facilitating the migration of basophils to inflamed tissue^6^. Thus, cytokine production and the accumulation of ILC2 and basophils can occur in the same phase of inflammation, and ILC2 and basophils accumulate in close proximity to each other^31^.

The characteristic ocular phenotype of IL33tg mice strongly suggests that IL-33 is crucial for the development of AKC. Epithelial IL-33 might stimulate resident-ILC2 and proliferates and activates ILC2-producing IL-5, IL-13 and chemokines, and thereby induce inflammatory processes in the conjunctiva and the cornea with eosinophils, basophils and mast cells. However, further studies are required to understand the mechanisms for AKC. As a novel mouse model for AKC with corneal impairment, IL33tg mice may be useful in research on corneal involvement in AKC and for the development of new therapeutic approaches to AKC.

## Methods

### Mice

All studies involving animals were reviewed and approved by the Animal Use and Care Committee of the Hyogo College of Medicine and were designed in accordance with the International Guiding Principles for Biomedical Research Involving Animals published by the Council for the International Organization of Medical Science. The mouse line hK14mIL33tg was generated and grown as described previously^7^. All mice used in this study were maintained under SPF conditions.

### Materials

Fluorescence-labeled antibodies for Siglec-F were purchased from BD Biosciences (San Jose, CA); those for B220, CD3, CD4, CD8, CD45, DX5, Gr-1, NK1.1, Sca-1, Thy-1.2 and IL-5 (TRFK5) were from BioLegend (San Diego, CA); those for FcεR1 (MAR-1), GATA3 (TWAJ), IL-13 (eBio13A) and Rat IgG1 (eBRG1), control antibody, were from e-Biosciences (San Diego, CA). Fluorescence-labeled antibody for ST2 (DJ8) was from MD Biosciences (St Paul, MN). For corneal fluorescein staining, Fluores^®^ ocular examination test papers (Showa Yakuhin Kako, Tokyo, Japan) were used according to the manufacturer’s instructions.

### Preparation of mouse conjunctiva and cornea

Dr. Yuka Hosotani and Dr. Hiroto Ishikawa, certified ophthalmic surgeons at Hyogo College of Medicine Hospital, prepared mouse ocular specimens under the microscope (Zeiss Stemi 2000-C stereo microscope). Ocular tissues were dissected using micro-scissors and forceps, a full thickness of the cornea was separated using a 2.0-mm trephine (Inami, Toyo, Japan), and conjunctiva was carefully separated from surrounding sclera and palpebra. These specimens were used for gene expression assay and flow cytometry.

### Quantitative real-time polymerase chain reaction (qPCR)

Total RNA of mouse tissues was prepared using an RNeasy Micro Kit (Qiagen, Hilden, Germany) according to the manufacturer’s instructions. A TaqMan^®^ RNA-to-Ct Kit (Applied Biosystems, Foster City, CA) and an ABI7900HT sequence detection system (Applied Biosystems) were used for qPCR. The expression of *Gapdh* that encodes glyceraldehyde-3-phosphate dehydrogenase was used as an internal standard for qPCR. The probes for qPCR were obtained from Applied Biosystems Assays-on-Demand. The product numbers of the probes for *Il1b*, *Il4*, *Il5*, *Il6, Il13*, *Il33*, *Prg2*, *Mmcp8, Ifng, Tnfa* and *Gapdh* were Mm00434228_m1, Mm00445259_m1, Mm00439646_m1, Mm00446190_m1, Mm00434204_m1, Mm00505403_m1, Mm01336479_m1, Mm00484933, Mm01168134_m1, Mm00443258_m1 and Mm99999915_g1, respectively. The abundance of each target transcript relative to the internal control was assessed according to the manufacturer’s instructions.

### Flow Cytometry

Cell suspensions from mouse tissue were prepared as described previously^7^. The method was modified as follows for corneal cells: Homogenized corneal sheets were incubated in 4 ml RPMI 1640 containing 1% fetal calf serum, 85 µg/ml Liberase^TM^ (Roche, Basel, Switzerland) and 0.01% DNase I (Roche) at 37 °C for 60 min. Cells were stained with each antibody and were examined using a flow cytometer LSRFortessa (BD Biosciences) or LE-SP6800Z (Sony, Tokyo, Japan). The classification of cells is as follows: Lineage-markers (B220, CD3, CD4, CD8, Gr-1, NK1.1, Siglec-F, DX5, FcεRI)^−^CD45^+^Sca-1^+^ST2^+^ cells: ILC2 cells. B220^−^CD3^−^CD4^−^CD8^−^Gr-1^−^NK1.1^−^Siglec-F^−^CD45^+^DX5^+^FcεRI^+^ cells: basophils. For intracellular cytokine staining, cornea cells were incubated in culture medium for 4 h in the presence of monensin with phorbol 12-myristate 13-acetate/ionomycin, and then surface antigens were stained in the presence of monensin. Following the fixation and permeabilization of the cells using a Fixation and Permeabilization Solution Kit (BD Biosciences), cells were stained with anti–IL-5, IL-13 or control rat IgG1 antibody. For intracellular GATA3 staining, cornea cells were stained using the Foxp3/Transcription Factor Staining Buffer Set (e-Biosciences, cat 00-5523).

### Tissue staining and immunofluorescence

Excised eye specimens were fixed with 4% (wt/vol) paraformaldehyde and then embedded in paraffin. The tissues were sectioned at 4-μm thickness, and deparaffinized sections were subjected to hematoxylin and eosin (H&E) staining or immunohistochemistry. Immunofluorescence for IL-33 was as described previously^7^. In brief, sections were incubated with an affinity-purified rabbit anti-mouse IL-33 polyclonal antibody, and bound antibodies were visualized with a biotinylated goat anti-rabbit IgG antibody (Vector Laboratories) and a Streptavidin, Alexa Fluor^®^ 594 conjugate (Invitrogen, Carlsbad, CA). Immunofluorescence for mMCP-8 was as described previously^9^. Sections were incubated with anti-mMCP-8 antibody (clone TUG8, BioLegend), and bound antibodies were visualized with a Cy3-conjugated donkey anti-rat IgG antibody (WAKO, Osaka, Japan). Following mounting with a ProLong^®^ Diamond Antifade with DAPI (Life Technologies, Gaithersburg, MD), fluorescence images were recorded using a confocal laser scanning microscope LSM780 (Carl Zeiss MicroImaging, Thornwood, NY).

### Collection of lacrimal fluid

Ten µL of phosphate-buffered saline (PBS) was introduced onto the ocular surface by a pipette^32^. The solution was then collected with a pipette from the tear meniscus.

### Enzyme linked immunosorbent assay (ELISA)

The concentrations of cytokines and chemokines in lacrimal fluid were measured using a Bio-Plex Protein Array System (Bio-Rad, Hercules, CA). This array allows the detection of multiple analyses in a small volume sample.

### Statistical analyses

Statistical analyses were performed using GraphPad Prism 6.0 (GraphPad Software, San Diego, CA). P-values < 0.05 were considered as significant differences.

## Electronic supplementary material

Supplementary Information
