# Supplementary material for: Expression of IL-33 in ocular surface epithelium induces atopic keratoconjunctivitis with activation of group 2 innate lymphoid cells in mice
Source: Sci Rep. 2017 Aug 30;7:10053. doi: 10.1038/s41598-017-10227-y (PMC5577277; doi:10.1038/s41598-017-10227-y)
Supplement: Supplementary file 1 — Supplementary Information [file 41598_2017_10227_MOESM1_ESM.pdf]

Supplementary Information

Expression of IL-33 in ocular surface epithelium induces atopic keratoconjunctivitis with activation of group 2 innate lymphoid cells in mice

Yasutomo Imai[1+], Yuka Hosotani[2+], Hiroto Ishikawa[2], Koubun Yasuda[3], Makoto Nagai[1], Orie Jitsukawa[1], Fumi Gomi[2], Kenji Nakanishi[3], Tomohiro Yoshimoto[3], Takahiro Nakamura[4], and Kiyofumi Yamanishi[1*]

[1]Department of Dermatology, [2]Department of Ophthalmology, [3]Department of Immunology, Hyogo College of Medicine, Nishinomiya 663-8501, Japan; [4]Department of Frontier Medical Science and Technology for Ophthalmology, Kyoto Prefectural University of Medicine, Kamigyo-ku, Kyoto 602-0841, Japan

[+] equally contributed.

[*] Correspondence should be addressed to:
Kiyofumi Yamanishi, M.D, Ph.D.
Department of Dermatology, Hyogo College of Medicine
1-1, Mukogawa-cho, Nishinomiya, Hyogo, 663-8501 Japan
Tel: +81 798 45 6653
FAX: +81 798 45 6651
E-mail: kyamanis@hyo-med.ac.jp

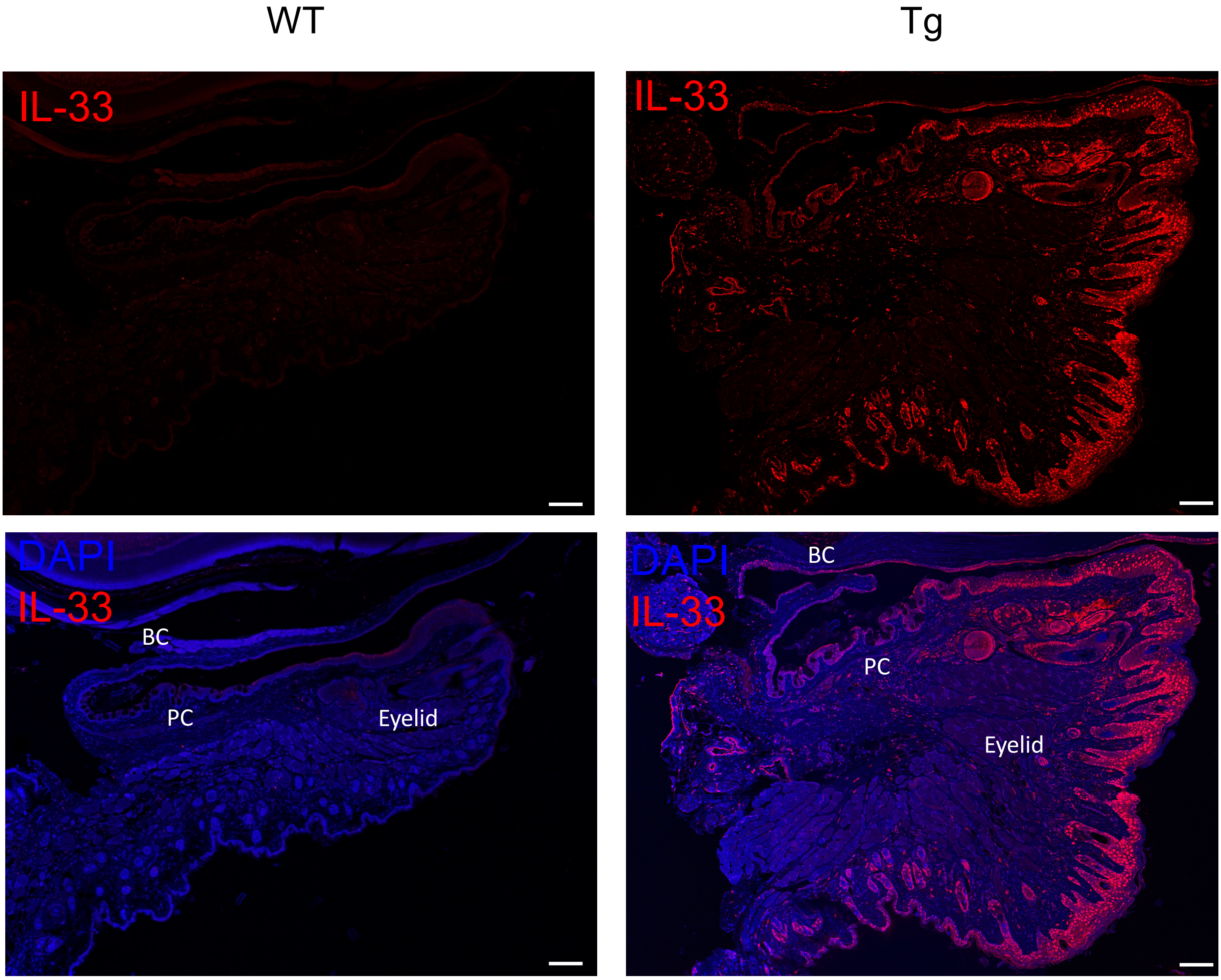

Supplementary Figure 1. Immunofluorescence of IL-33 in the ocular surface epithelium and the whole eyelid of 20- to 30-week-old WT and IL33tg mice. Intense staining of IL-33 was evident in nuclei of the conjunctival epithelium and the epidermis in IL33tg mice (Tg). BC, bulbar conjunctiva. PC, palpebral conjunctiva. Bars, 100 μm. Data are representative of at least five mice and two independent experiments.

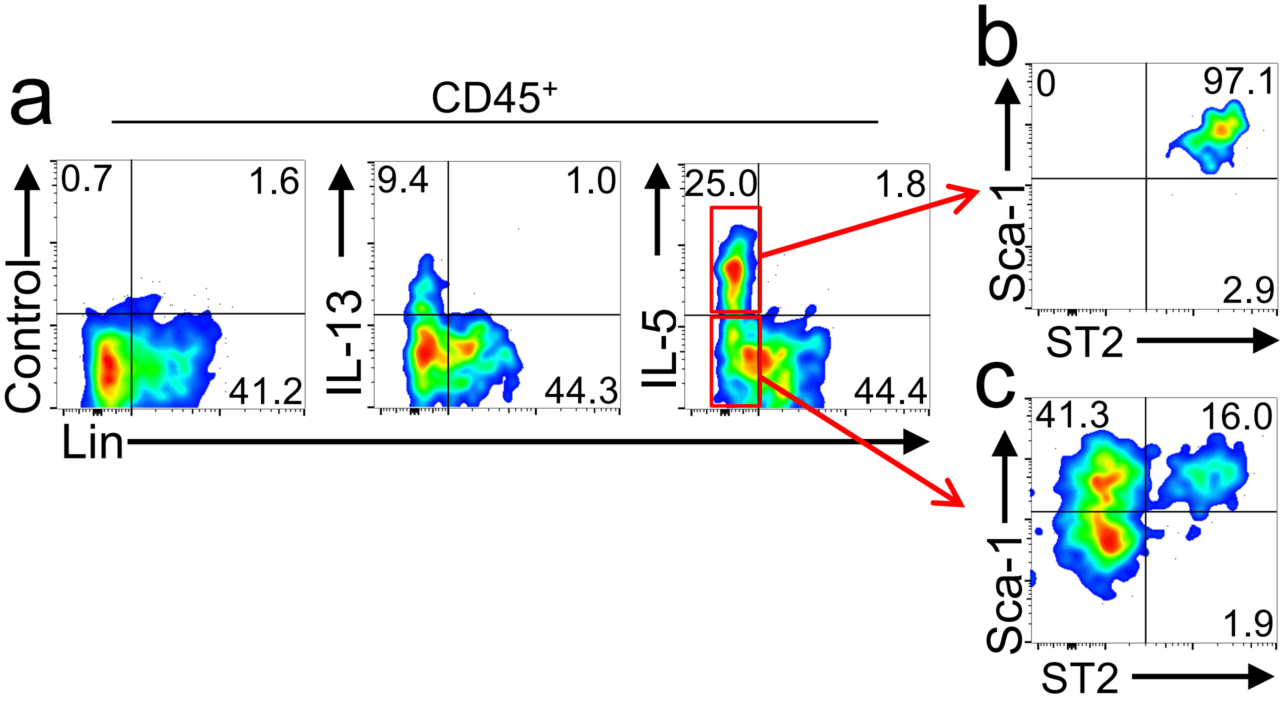

Supplementary Figure 2. ILC2 are a major source for IL-5 and IL-13 in the lesional cornea.

(a) The intracellular flow cytometry for IL-5 and IL-13. ILC2 from 20- to 30-week-old IL33tg mouse cornea expressed high levels of IL-5 and IL-13. The cells were gated into $CD45^+$ fraction (a), $CD45^+Lin^-IL\text{-}5^+$ fraction (b) and $CD45^+Lin^-IL\text{-}5^-$fraction (c). Note that $IL\text{-}5^+$ population is $Sca\text{-}1^+$ $ST2^+$ ILC2 (b). The numbers indicate the percentage of cells in each quadrant. Similar results were obtained in three independent experiments.

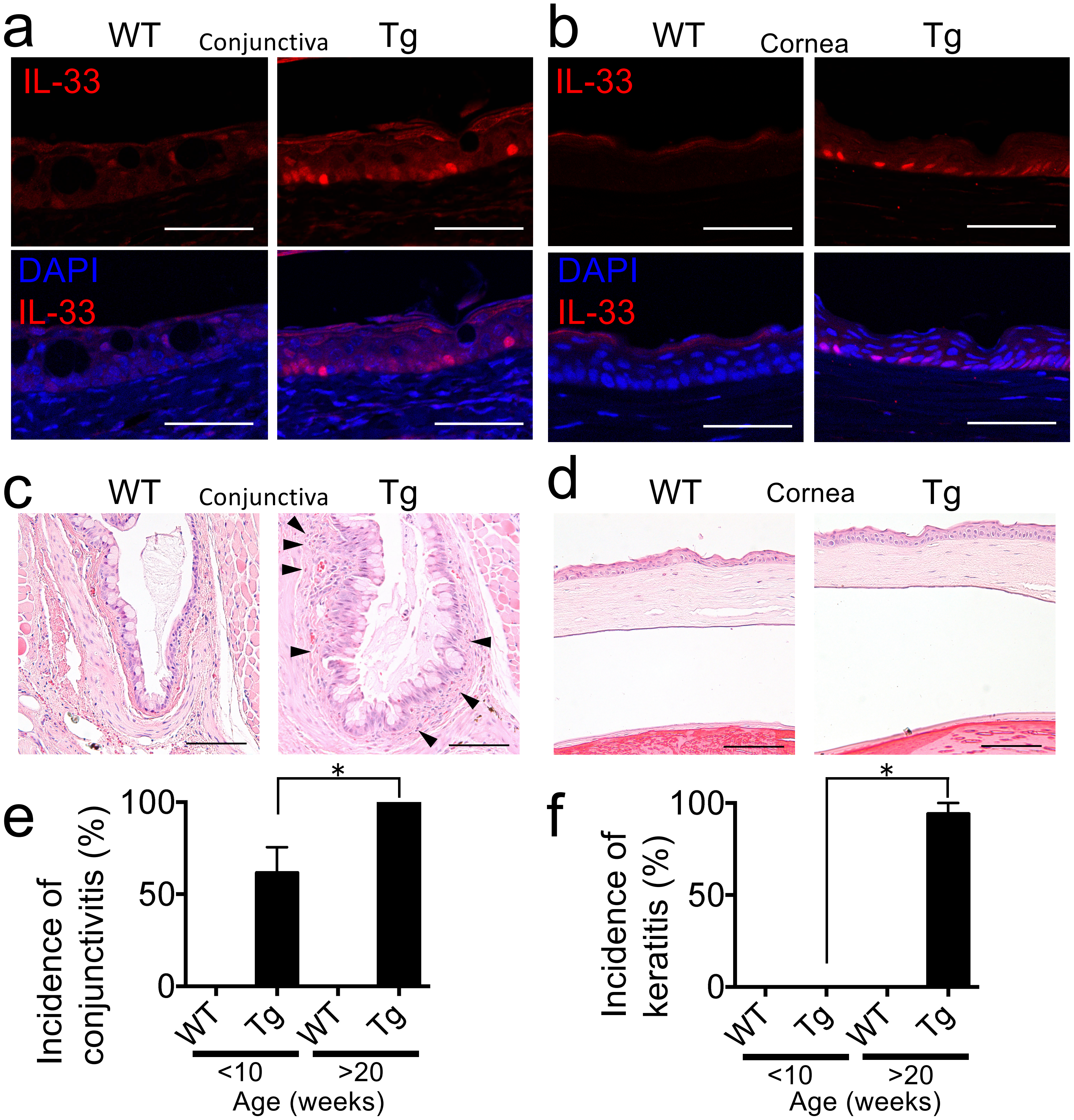

Supplementary Figure 3. Ocular phenotype of pre-disease in 8- to 10-week-old IL-33Tg mice. (a, b) Immunofluorescence of IL-33 in the ocular surface epithelium of 8- to 10-week-old WT and IL33tg mice (Tg). Intense staining of IL-33 was evident in nuclei of the conjunctival epithelium (a) and corneal epithelial cells (b) in IL33tg mice. Bars, 50 μm. (c, d) H&E staining of 8- to 10-week-old WT and IL33tg mouse (Tg) cornea and conjunctiva. Mild eosinophilic infiltration (arrowheads) was apparent in the conjunctiva (c). In contrast, cornea of IL33tg mice was intact (d). Panels are representative of at least five mice and two independent experiments. Bars, 100 μm. (e, f) Incidence of spontaneous conjunctivitis (e) and keratitis (f) were examined by histological confirmation (H&E staining). Eight- to 10-week-old (<10) WT mice, n = 15; 8- to 10-week-old (<10) IL33tg mice (Tg), n = 13; 20- to 30-week-old (>20) WT mice, n = 11; 20- to 30-week-old (>20) IL33tg mice (Tg), n = 17. *$P < 0.05$ (Mann-Whitney test).

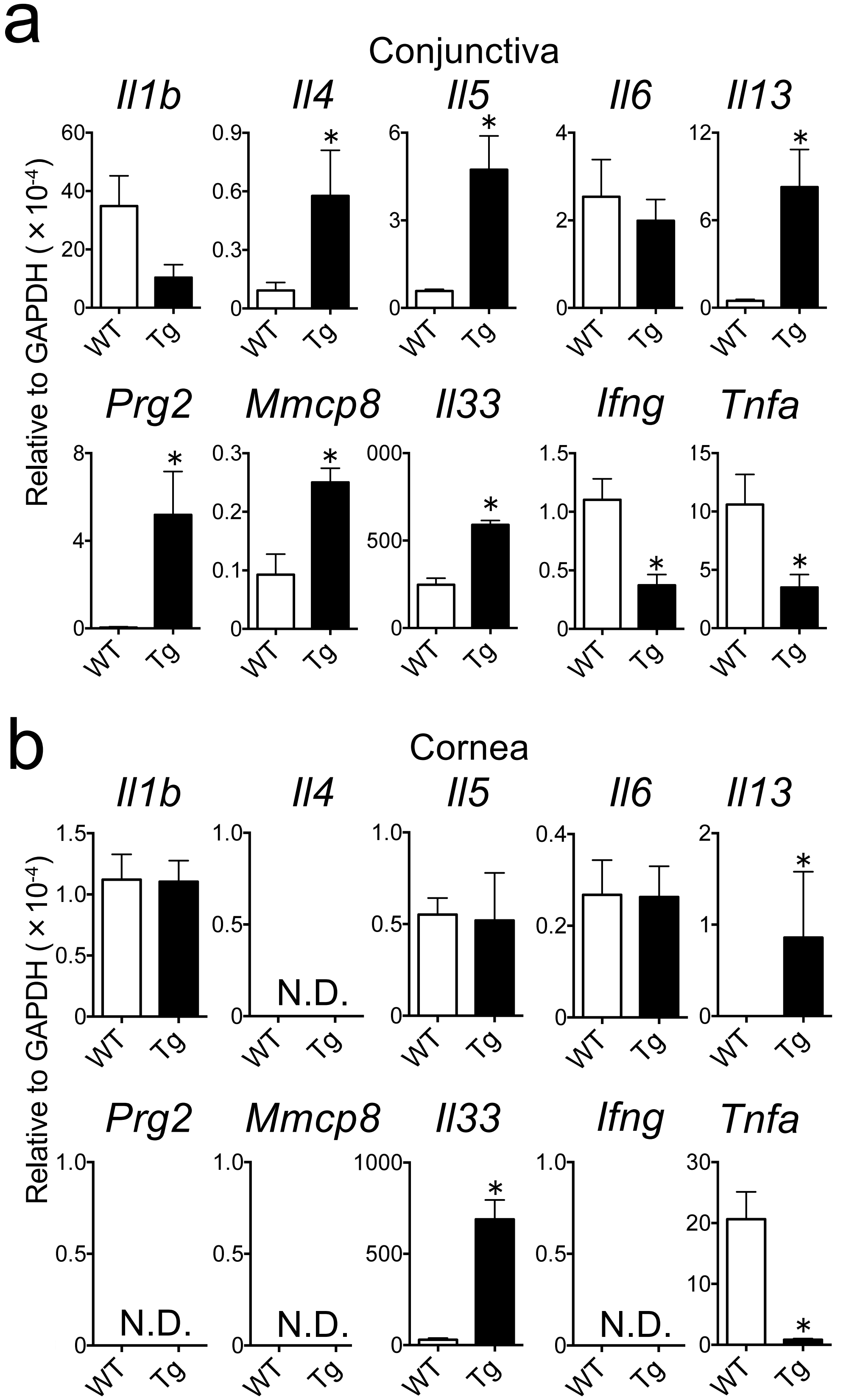

Supplementary Figure 4. The gene expressions of Th2 cytokines, eosinophil granule major basic protein (Prg2) and mouse mast cell protease 8 (Mmcp8) in the conjunctiva and cornea of 10-week-old IL33tg mice. Note that *Il4*, *Il5*, *Prg2* and *Mmcp8* were increased in the conjunctiva (a), but not in the cornea (b) of 10-week-old IL33tg mice (Tg). Data are expressed as means ± SEM (n = 5), *P < 0.05 (Mann-Whitney test). N.D., not detected.
